# Editorial: Extracellular vesicles signaling in embryogenesis and morphogenesis

**DOI:** 10.3389/fcell.2026.1803773

**Published:** 2026-02-18

**Authors:** Cornelia M. Wilson, Carol M. Trim, Alex George

**Affiliations:** 1 Life Sciences Industry Liaison Lab, Natural Applied Sciences, School of Science, Psychology, Arts and Humanities, Computing, Engineering and Sport, Canterbury Christ Church University, Sandwich, United Kingdom; 2 Jubilee Centre for Medical Research, Jubilee Mission Medical College and Research Institute, Thrissur, Kerala, India

**Keywords:** development, embryogenesis, extracellular vesicles, morphogenesis, reproduction

Extracellular vesicles (EVs) have emerged as fundamental mediators of intercellular communication across diverse biological systems, influencing development, tissue homeostasis, and disease progression (Tkach and Théry, 2016; van Niel et al., 2018). This Research Topic, *Extracellular Vesicles in Embryogenesis and Morphogenesis* was assembled to bring together mechanistic, translational, and conceptual advances that highlight the central role of EVs in coordinating complex biological processes across reproductive and developmental contexts.

**FIGURE 1:**
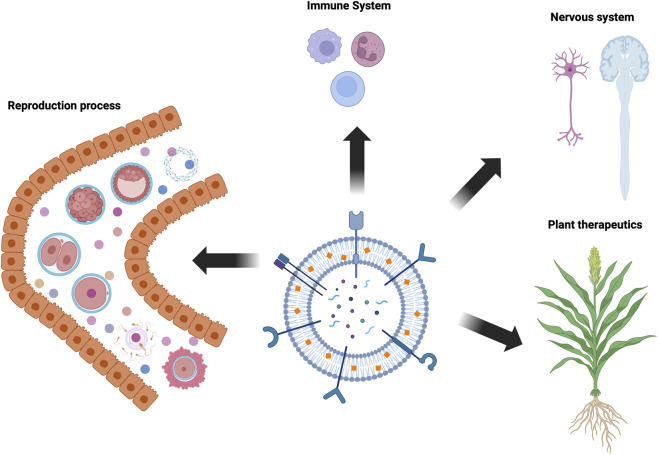
Overview of extracellular vesicle (EV) roles across embryogenesis and morphogenesis. Extracellular vesicles act as dynamic mediators of intercellular communication across biological systems. In reproductive biology, EVs regulate gametogenesis, placentation, and embryo–maternal communication, while also serving as minimally invasive biomarkers of placental dysfunction. Mechanistic studies highlight EV-mediated control of trophoblast, endothelial, and immune cell behaviour at the maternal–fetal interface. Beyond reproduction, exosomes contribute to neural development by modulating axon guidance and circuit formation. Emerging paradigms, including plant-derived extracellular vesicles, offer scalable and biocompatible alternatives that may support future translational and therapeutic applications.

From its original conception, this Research Topic was motivated by a growing recognition that EV-mediated signalling represents an additional and underexplored layer of regulation operating alongside classical morphogen gradients and contact-dependent signalling during embryogenesis and tissue patterning (Wolpert, 1969; Rogers and Schier, 2011). Rather than acting as passive by-products of cellular activity, EVs are increasingly understood as structured, information-rich entities capable of integrating spatial and temporal cues across tissues, developmental stages, and physiological states. The contributions assembled here collectively reflect this shift in perspective, positioning EVs as active organisers of developmental and reproductive processes (Figure 1).

A unifying theme across the contributions is the role of EVs as dynamic conveyors of molecular information, integrating spatial and temporal regulation across tissues. Within reproductive biology, EVs are increasingly recognised as key regulators of gametogenesis, placentation, embryo–maternal communication, and pregnancy maintenance. The articles in this Research Topic collectively demonstrate how EV cargo composition reflects tissue state and function, and how dysregulation of EV-mediated signalling can contribute directly to reproductive pathologies Pan et al.

One contribution provides a comprehensive review of extracellular vesicles in reproductive medicine, framing the field’s evolution from an animal-derived EV paradigm toward emerging plant-derived extracellular vesicle platforms Pan et al. This work situates EV research within a broader sustainability and translational framework, critically evaluating barriers to clinical implementation including heterogeneity, scalability, immunogenicity, and sourcing constraints while proposing plant-derived EVs as a potentially transformative and ethically favourable alternative for future precision therapies. This perspective aligns with broader efforts to overcome long-standing translational bottlenecks in EV research (van Niel et al., 2018).

Complementing this conceptual overview, original research within the Research Topic demonstrates the diagnostic potential of placental EVs as minimally invasive indicators of placental dysfunction Lee et al. Using a genetically defined mouse model, this study shows that tissue-derived placental EVs faithfully capture mid-gestational defects in placental development, angiogenesis, and immune regulation. Proteomic and miRNA profiling revealed EV cargo signatures associated with adverse pregnancy outcomes, reinforcing the concept that EVs retain a molecular fingerprint of their tissue of origin and may serve as early biomarkers of compromised placental function—an idea increasingly central to EV biology and liquid biopsy strategies (Peinado et al., 2017).

Further mechanistic insight into placental EV function is provided by work examining the regulatory effects of placental EVs on trophoblast and endothelial cell behaviour (Bai et al.). This study highlights how placental EVs coordinate trophoblast differentiation, migration, and invasion while concurrently modulating endothelial function—processes essential for spiral artery remodelling and successful establishment of the maternal–fetal interface. These findings reinforce emerging models in which EVs fine-tune developmental signalling pathways rather than simply amplifying them, adding spatial precision to morphogenetic processes.

Extending beyond reproductive systems, this Research Topic also includes a review exploring the emerging role of exosomes in axon guidance during central nervous system development Liu and Teng. By positioning exosomes as mobile signalling platforms that operate alongside canonical guidance cues including Wnt, Hedgehog, and semaphorin pathways this work broadens the relevance of EV biology to neural circuit formation and neurodevelopmental disorders. Importantly, it reinforces the concept that EV-mediated communication represents a conserved mechanism by which developmental information is distributed across tissues and over distance (Gross et al., 2012; Matusek et al., 2014).

Taken together, the contributions to this Research Topic underscore the versatility of extracellular vesicles as integrative regulators of development and disease. Across reproductive and neural contexts, EVs emerge not merely as passive carriers of molecular cargo, but as active biological agents shaping cellular behaviour, tissue architecture, and physiological outcomes. At the same time, this Research Topic highlights persistent challenges in the field—including the need for methodological standardisation, improved cargo characterisation, and rigorous functional validation—while pointing toward innovative strategies that may accelerate translational progress.

We hope that this Research Topic provides a coherent snapshot of current advances in extracellular vesicle biology, stimulates cross-disciplinary dialogue, and encourages further exploration of EV-based diagnostics and therapeutics. We thank all contributing authors and reviewers for their valuable contributions and critical insights, and we anticipate that the work presented here will inform and inspire future research at the interface of extracellular vesicle biology, development, and medicine.
